# Reply: metrics to assess machine learning models

**DOI:** 10.1038/s41746-018-0063-z

**Published:** 2018-10-10

**Authors:** Alvin Rajkomar, Andrew M. Dai, Mimi Sun, Michaela Hardt, Kai Chen, Kathryn Rough, Jeffrey Dean

**Affiliations:** 1 Google LLC, Mountain View, CA USA; 2 University of California, San Francisco, San Francisco, CA USA

**Keywords:** Health services, Outcomes research

We thank Prof. Pinker for bringing up important points on how to assess the performance of machine learning models. The central finding of our work is that a machine learning pipeline operating on an open-source data-format for electronic health records can render accurate predictions across multiple tasks in a way that works for multiple health systems. To demonstrate this, we selected three commonly used binary prediction tasks, inpatient mortality, 30-day unplanned readmission, and length of stay, as well as the task of predicting every discharge diagnosis. The main metric we used for the binary predictions was the area-under-the-receiver-operator curve (AUROC).

We would first like to clarify a few issues. We would highlight in our results section that we did report the number-needed-to-evaluate or work-up to detection ratio for the inpatient mortality model and baseline model, which is (1/PPV) and commonly accepted as a clinically relevant metric.^1^

Also, as described in the “Study Cohort” section, we only included hospitalizations of 24 h or longer, and Table 1 reports the inpatient mortality rates of the hospitals to be approximately 2% in that cohort. This should not be confused with 2.3% of patients dying within 24 h.

**Table 1:** Area under the precision-recall curves for various predictions

	Hospital A	Hospital B
Inpatient Mortality, 24 h after admission, AUPRC		
Deep learning model	0.41 (0.34–0.48)	0.42 (0.37–0.48)
Baseline (aEWS) model	0.24 (0.18–0.32)	0.25 (0.20–0.30)
Full feature, enhanced baseline	0.32 (0.25–0.39)	0.29 (0.25–0.35)
30 day unplanned readmission at discharge, AUPRC		
Deep Learning Model	0.28 (0.26–0.30)	0.37 (0.36–0.40)
Baseline (mHospital) at discharge	0.20 (0.19–0.22)	0.28 (0.26–0.29)
Full feature, enhanced baseline	0.25 (0.24–0.28)	0.34 (0.33–0.36)
Length of stay at least 7 days, AUPRC		
Deep learning model	0.67 (0.65–0.69)	0.66 (0.65–0.68)
Baseline (Liu) model	0.47 (0.44–0.49)	0.48 (0.46–0.50)
Full feature, enhanced baseline	0.63 (0.61–0.66)	0.63 (0.61–0.64)

Prof. Pinker states that the public could be mislead by the way the mainstream media had reported the results of our paper. We observed that many reports incorrectly conflated accuracy with AUROC. We take our responsibility seriously to clearly explain our results to a more general audience and had simultaneously released a public blog post.^2^ In that post, we talked explicitly about the AUROC: “The most common way to assess accuracy is by a measure called the area-under-the-receiver-operator curve, which measures how well a model distinguishes between a patient who will have a particular future outcome compared to one who will not. In this metric, 1.00 is perfect, and 0.50 is no better than random chance, so higher numbers mean the model is more accurate.”

We agree that the AUROC has its limitations, although we would note that no single metric conveys a complete picture of the performance of a model. The AUROC has an advantage of being a commonly reported metric in both clinical and recent machine-learning papers.^3^ We did caution in our manuscript that direct comparison of AUROCs from studies using different cohorts is problematic.^4^

However, we do agree that the area under the precision-recall curve (AUPRC) is relevant for prediction tasks and can be particularly helpful with clinical tasks with high class imbalance.

Therefore, we report the AUPRC for each of the binary prediction tasks for the primary models reported in the manuscript, the clinical baselines, and the enhanced-baselines that we described in the supplemental materials (Table 1). The confidence intervals are calculated by stratified bootstrapping of the positive and negative classes, as is common for this metric.^5^ It is worth noting that the models evaluated here were tuned to optimize the AUROC, and it is well-known that a model tuned for optimizing AUROC does not necessarily optimize AUPRC (and vice-versa). The size of the test set (9624 for Hospital A and 12,127 for Hospital B) limits the power to make comparisons between models, although the point-estimates are higher for the deep learning models for each case.
